# Mixed adeno(neuro)endocrine carcinoma arising from the ectopic gastric mucosa of the upper thoracic esophagus

**DOI:** 10.1186/1477-7819-11-218

**Published:** 2013-09-04

**Authors:** Toshihiro Kitajima, Sachiko Kaida, Seigi Lee, Shusuke Haruta, Hisashi Shinohara, Masaki Ueno, Koichi Suyama, Yasunori Oota, Takeshi Fujii, Harushi Udagawa

**Affiliations:** 1 Department of Digestive Surgery, Toranomon Hospital, Tokyo, Japan; 2 Department of Medical Oncology, Toranomon Hospital, Tokyo, Japan; 3 Department of Pathology, Toranomon Hospital, Tokyo, Japan

**Keywords:** Adenocarcinoma, Ectopic gastric mucosa, Esophagus, Mixed adenoendocrine carcinoma

## Abstract

We report a case of mixed adenoendocrine carcinoma of the upper thoracic esophagus arising from ectopic gastric mucosa. A 64-year-old man who had been diagnosed with an esophageal tumor on the basis of esophagoscopy was referred to our hospital. Upper gastrointestinal endoscopy revealed the presence of ectopic gastric mucosa and an adjacent pedunculated lesion located on the posterior wall of the upper thoracic esophagus. Subtotal esophagectomy with three-field lymph node dissection was performed. A microscopic examination revealed that there was a partially intermingling component of neuroendocrine carcinoma adjacent to a tubular adenocarcinoma which was conterminous with the area of the ectopic gastric mucosa. Although the tubular adenocarcinoma was confined to the mucosa and submucosa, the neuroendocrine carcinoma had invaded the submucosaand there was vascular permeation. Each component accounted for 30% or more of the tumor, so the final histopathological diagnosis was mixed adenoendocrine carcinoma of the upper thoracic esophagus arising from ectopic gastric mucosa. Adjuvant chemotherapy was not performed, because the postoperative tumor stage was IA. The patient was well and had no evidence of recurrence 16 months after surgery.

## Background

Most esophageal carcinomas are squamous cell carcinomas or adenocarcinomas arising from Barrett’s epithelium, whereas adenocarcinomas derived from the esophageal glands or ectopic gastric mucosa (EGM)are rare. These cases arise mostly in the cervical or upper thoracic esophagus [1]. Moreover, gastrointestinal tumors displaying both exocrine and neuroendocrine differentiation are uncommon. To the best of our knowledge, esophageal mixed adenoneuroendocrine carcinoma (MANEC) [2] arising from EGM is extremely rare. We report a case of MANEC in the upper thoracic esophagus arising from EGM and also provide a review of the pertinent literature.

## Case presentation

**Figure 1:**
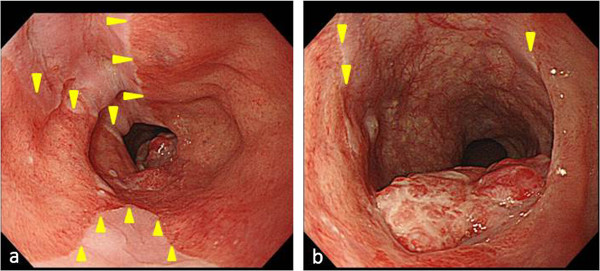
**Upper gastrointestinal endoscopic findings regarding the pedunculated tumor. (a)** Endoscopy revealed ectopic gastric mucosa 19 to 21 cm distal from the incisors on the oral side of the pedunculated tumor (arrowheads). **(b)** A pedunculated lesion was located on the posterior wall of the upper thoracic esophagus 21 to 23 cm distal from the incisors, adjacent to the area of ectopic gastric mucosa (arrowheads).

A 64-year-old Japanese man who had been diagnosed with an esophageal tumor during a screening esophagoscopy was referred to our hospital. He had a history of Miles operation for rectal cancer 11 years earlier and partial hepatectomy and right lateral lymph node dissection for metastasis from rectal cancer 6 years prior to his presentation at our hospital. He had been smoking 20 cigarettes per day since his 20s, consumed alcohol only on social occasions and was not a regular habitual drinker. A laboratory analysis showed no abnormalities in any parameters, including the levels of tumor markers such as squamous cell carcinoma antigen, carcinoembryonic antigen and carbohydrate antigen 19–9 (CA 19–9). An upper gastrointestinal endoscopy demonstrated EGM 19 to 21 cm distal from the incisors (Figure 1a) and a pedunculated lesion located on the posterior wall of the upper thoracic esophagus 21 to 23 cm distal from the incisors (Figure 1b), which was adjacent to the area of the EGM. A biopsy taken from the pedunculated lesion revealed well-differentiated tubular adenocarcinoma. Endoscopic ultrasound revealed that the tumor had invaded the submucosa. Computed tomography detected abnormal thickening at the posterior wall of the upper thoracic esophagus without any metastases to the lymph nodes or other organs.

**Figure 2:**
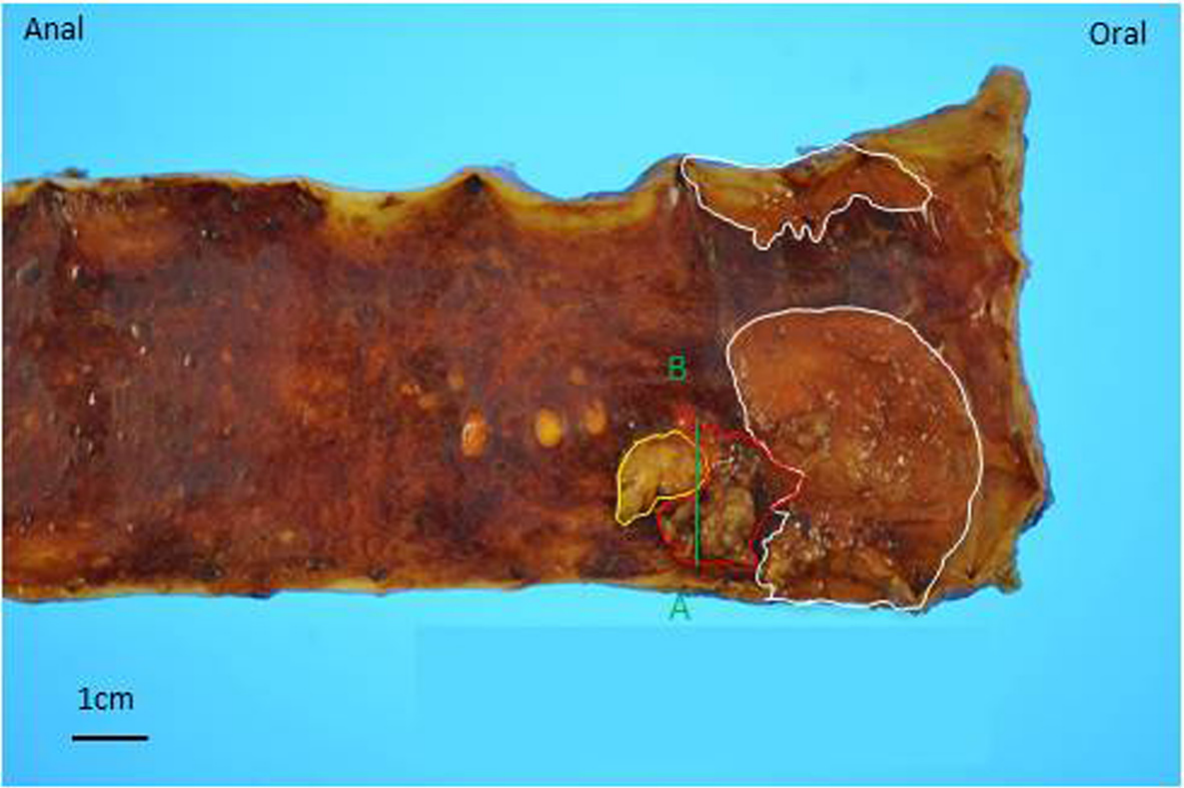
**Gross findings of the resected specimen.** The pedunculated tumor, which measured 17 × 15 mm, is indicated within the red outline adjacent to a rough area 36 × 30 mm in size, which is indicated within the white outlines. The area within the yellow outline was composed of neuroendocrine carcinoma. The relationship between the NEC and adenocarcinoma components was confirmed on the green line in Figure 3.

**Figure 3:**
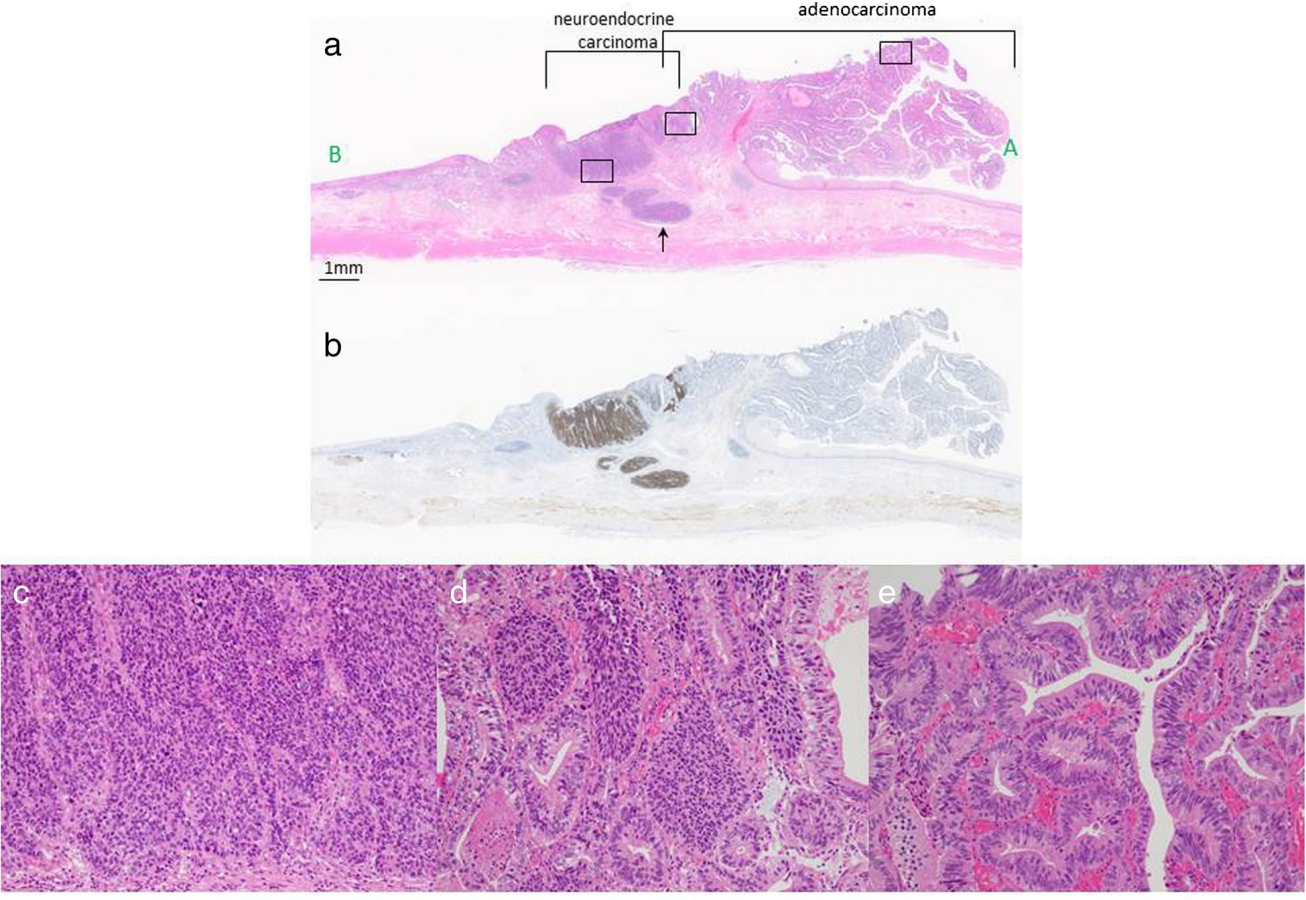
**Histopathologic findings. (a)** A low-power view of the line (from A to B) shown in Figure 2. The neuroendocrine carcinoma (NEC) and adenocarcinoma components of the tumor were adjacent and partially intermingled. The NEC component had invaded the submucosa with vascular permeation (arrow). **(b)** The NEC component showed a positive response for CD56 staining (immunohistochemical stain). **(c)** A magnified view of the squared area at the left in **(a)**. The NEC component showed a solid and trabecular pattern, and the tumor cells had elongated hyperchromatic nuclei and scant cytoplasm (hematoxylin and eosin stain (H&E); original magnification, ×117). **(d)** A magnified view of the squared area in the middle in **(a)**. A borderline area of NEC and well-differentiated adenocarcinoma showed a histological transition (H&E; original magnification, ×117). **(e)** A magnified view of the squared area at the right in **(a)** showing well-differentiated tubular adenocarcinoma confined to the mucosa and submucosa (H&E; original magnification, ×117).

The patient was diagnosed with primary adenocarcinoma arising from EGM in the upper thoracic esophagus. He underwent radical esophagectomy with three-field lymphadenectomy. Surgical reconstruction was performed through the posterior mediastinal route using a gastric conduit, followed by esophagogastrostomy through a cervical incision. Grossly, the pedunculated tumor, which measured 17 × 15 mm (area within the red outline in Figure 2) was seen adjacent to a rough area 36 × 30 mm in size (areas within white outlines in Figure 2). Histopathologically, the pedunculated tumor consisted of well-differentiated tubular adenocarcinomaconfined within the submucosa (Figure 3a and e). The adjacent solid and trabecular component (corresponding to the area within the yellow outline in Figure 2) was composed of tumor cells showing elongated hyperchromatic nuclei and scant cytoplasm (Figure 3a and c), which were immunoreactive for CD56 (Figure 3b) and synaptophysin, confirming the diagnosis of neuroendocrine carcinoma (NEC). Additionally, vascular permeation of NEC was seen in the submucosal vein (Figure 3a, arrow). There was a histological transition between the NEC and tubular adenocarcinoma (Figure 3d), and the area of adenocarcinoma was conterminous with the EGM (corresponding to the area within the white outlines in Figure 2). The NEC and adenocarcinoma components accounted for at least 30% of the tumor lesion, respectively, confirming the diagnosis of MANEC. None of the 79 lymph nodes widely dissected, as was defined in our previous report [3], had metastases, and no lymphatic invasion was noted. The patient was diagnosed with stage IA (pT1bN0M0) disease according to the edition of the American Joint Committee on Cancer and the International Union Against Cancer TNM classification system [4]. Postoperatively, bilateral recurrent laryngeal nerve injury was noted, and a tracheostomy was placed. Six months after the surgery, resection of the pyloric ring and diversion of the gastric conduit was performed in a Roux-en-Y fashion to prevent repetitive aspiration of regurgitant. For these reasons, it took approximately 8 months for the patient to safely resume oral intake. At the time of this writing, the patient has been doing well for 16 months, with no evidence of recurrence.

### Discussion

Adenocarcinoma of the esophagus originates mostly from Barrett’s epithelium in the lower esophagus. Primary adenocarcinoma of the cervical and upper thoracic esophagus is rare and is considered to be derived from the esophageal glands (mucosal or submucosal) or from EGM [1]. The reported incidence of adenocarcinoma in the upper esophagus has been found to account for only 1% to 2% of all malignant esophageal tumors [5], and 33 cases of adenocarcinoma derived from EGM have been described worldwide in the published literature to date (Table 1). Among the 2,237 surgical cases of esophageal cancer in our hospital during the past 40 years, only 2 were primary adenocarcinomas arising from EGM. EGM often occurs in the upper esophagus. Its incidence has been reported to range from 2% to 14%, but these data have been increasing due to developments in endoscopic technology [6]. Although most patients with EGM are asymptomatic, some develop symptoms based on acid secretion from the EGM, such as dysphagia or a sore throat. Furthermore, EGM is sometimes accompanied by severe complications such as bleeding, perforation, stricture, tracheoesophageal fistula formation or webbing [7]. In our patient, it is noteworthy that the area of NEC existed adjacent to the area of tubular adenocarcinoma, which was conterminous with the EGM.

**Table 1:** **Summary of esophageal adenocarcinomas arising from ectopic gastric mucosa published in the literature (*N*** **= 34)**^**a**^

**Case**	**Reference**	**Year**	**Age**	**Sex**	**Histology**	**TNM**	**Treatment**	**Postoperative course**
1	Carrie [8]	1950	64	M	Adenocarcinoma	pT2NXM0	Resection of the upper esophagus	No recurrence (>1 yr)
2	Morson and Belcher [9]	1952	56	M	Adenocarcinoma	pT3N1M0	Esophagectomy	Unknown
3	Raphael *et al*. [10]	1966	69	M	Well- differentiated adenocarcinoma	unknown	Radiotherapy	Died (suicide) (2 mo)
4	Davis *et al*. [11]	1969	68	M	Mucinous adenocarcinoma	pT1(SM)NXMX	Radiotherapy + esophagectomy	No recurrence (7 mo)
5	Sakamoto *et al*. [12]	1970	64	M	Adenocarcinoma	pT2N0M0	Esophagectomy	Died (10 mo)
6	Jernstrom and Brewer [13]	1970	73	M	Poorly differentiated adenocarcinoma	pT3N0M0	Radiotherapy + esophagectomy	Died (4 mo)
7	Clemente [14]	1974	53	M	Adenocarcinoma	pT3	Esophagectomy	Recurrence (10 mo)
8	Danoff *et al*. [15]	1978	43	M	Poorly differentiated adenocarcinoma	cT4NXMX	Radiotherapy	Died (9 mo)
9	Goëau-Brissonnière et al. [16]	1985	38	M	Adenocarcinoma	pT3	Esophagectomy	No recurrence (31 mo)
10	Schmidt *et al*. [17]	1985	37	M	Adenocarcinoma	pT3	Esophagectomy	Died (4 mo)
11	Christensen and Sternberg [18]	1987	52	M	Poorly differentiated adenocarcinoma	pT2N1M0	Esophagectomy	Recurrence (25 mo)
12	Christensen and Sternberg [18]	1987	50	M	Moderately differentiated adenocarcinoma	pT3N1M0	Esophagectomy	Unknown
13	Ishii *et al*. [19]	1991	66	M	Moderately differentiated adenocarcinoma	pT3N1M0	Esophagectomy	No recurrence (20 mo)
14	Takagi *et al*. [20]	1995	70	M	Well- differentiated adenocarcinoma	pT1(SM)N0M0	Esophagectomy	Unknown
15	Sperling and Grendell [21]	1995	79	M	Poorlydifferentiated adenocarcinoma	cT4N0M0	Radiotherapy	Unknown
16	Pai *et al*. [22]	1997	60	M	Poorly differentiated adenocarcinoma	pT2N0M0	Surgery/radiochemotherapy	Recurrence (24 mo)
17	Berkelhammer *et al*. [23]	1997	71	M	Moderately differentiated adenocarcinoma	pT1(SM)N1M0	Esophagectomy	No recurrence (2 yr)
18	Lauwers *et al*. [24]	1998	57	F	Moderately differentiated adenocarcinoma	pT3N0M0	Esophagectomy + adjuvant radiotherapy	No recurrence (8 mo)
19	Klaase *et al*. [25]	2001	43	M	Poorly differentiated adenocarcinoma	pT4N1M0	Esophagectomy + adjuvant radiotherapy	Died (4 mo)
20	Pech *et al*. [26]	2001	77	M	Well-differentiated adenocarcinoma	cT1(SM)N0M0	Endoscopic mucosal resection	No recurrence (1 yr)
21	Noguchi *et al*. [27]	2001	73	M	Well-differentiated adenocarcinoma	cT1(SM)N0M0	Resection of the cervical esophagus	No recurrence (5 yr)
22	Chatelain *et al*. [28]	2002	61	M	Poorly differentiated adenocarcinoma	pT3NXM0	Esophagectomy	Died (15 mo)
23	Hirayama *et al*. [29]	2003	77	F	Well-differentiated adenocarcinoma	cT1(M)N0M0	Endoscopic mucosal resection	No recurrence (31 mo)
24	Balon *et al*. [30]	2003	61	M	Adenocarcinoma	pT3N0M0	Esophagectomy	Died (21 mo)
25	Abe *et al*. [31]	2004	50	M	Well-differentiated adenocarcinoma	pT1(SM)N0M0	Esophagectomy	No recurrence (18 mo)
26	von Rahden *et al*. [1]	2005	52	M	Moderately differentiated adenocarcinoma	cT3N1M0	Neoadjuvant chemoradiotherapy + surgery	No recurrence (36 mo)
27	Alrawi *et al*. [32]	2005	60	M	Moderately differentiated adenocarcinoma	pT1(SM)N0M0	Esophagectomy + adjuvant Chemoradiotherapy	No recurrence (6 yr)
28	Hoshino *et al*. [33]	2007	74	M	Papillary adenocarcinoma	pT3N0M0	Esophagectomy	No recurrence (5 mo)
29	Alagozlu *et al*. [34]	2007	57	M	Poorly differentiated adenocarcinoma	cT4N1M0	None	Died before treatment
30	Komori *et al*. [35]	2010	75	M	Moderately differentiated adenocarcinoma	cT2N1M0	Esophagectomy	No recurrence (42 mo)
31	Iitaka *et al*. [36]	2011	64	M	Poorly differentiated adenocarcinoma	pT1(M)N0M0	Esophagectomy	No recurrence (36 mo)
32	Akanuma *et al*. [37]	2013	57	M	Well-differentiated adenocarcinoma	pT2N0M0	Esophagectomy + chemoradiotherapy	No recurrence (4 yr)
33	Nonaka *et al*. [38]	2013	74	M	adenocarcinoma	unknown	Endoscopic submucosal dissection	Unknown
34	Present case	2013	64	M	Well-differentiated adenocarcinoma	pT1bN0M0	Esophagectomy	No recurrence (16 mo)

^a^The cases included in this table are those available in PubMed as of 5 August 2013. *TNM* tumor, node, metastasis.

The term *mixed exocrine*-*endocrine carcinoma* (MEEC), which was proposed by the World Health Organization (WHO) in its classification system of endocrine tumors, refers to a neoplasm with divergent exocrine and neuroendocrine differentiation [39]. In the latest WHO classification system published in 2010 [2], neuroendocrine neoplasms in the digestive system were reclassified as NET G1, NET G2, NEC and MANEC according to the degree of cellular differentiation and proliferative activity [40]. MEEC/MANEC is distinguished from carcinomas with focal neuroendocrine differentiation by at least two major diagnostic criteria: (1) extension of each component (at least 30%) and (2) structural features of neuroendocrine components as well-differentiated organoid or solid or diffuse growth patterns [41]. Several cases of MANECs of digestive organs have been reported to be detected in the colon, pancreas, gallbladder, biliary tract, stomach, ampulla, cecum and esophagogastric junction [42-64] (Table 2). Lewin proposed a classification of morphological patterns of the two components in MEEC/MANEC distinguishing (1) truly composite (or mixed) exocrine-endocrine tumors with both elements in more or less equal proportions, (2) amphicrine tumors with dual differentiation within the same cell and (3) collision tumors, in which two components are closely juxtaposed but not admixed [65]. According to this classification scheme, our present case was considered to be a composite (mixed) adenoendocrine carcinoma.

**Table 2:** **Summary of the cases of mixed adeno(neuro)endocrine carcinoma published in the English-language literature after 2010 (*N*** **= 47)**^**a**^

**Affected organ**	**Cases**	**Mean age (yr)**	**Sex (M/F)**	**SYN (+/−/unknown)**	**CGA (+/−/unknown)**	**CD56 (+/−/unknown)**
Colon [6,7]	13	71	9/4	13/0/0	13/0/0	0/0/13
Pancreas [39-46]	13	69	11/2	11/0/2	12/0/1	0/0/13
Gallbladder [47-50]	8	63	1/7	8/0/0	7/0/1	2/0/6
Biliary tract [46,51,52]	6	71	3/3	6/0/0	5/1/0	1/0/3
Stomach [53-56]	4	71	1/3	4/0/0	3/1/0	2/1/1
Ampulla [57]	1	81	1/0	1/0/0	1/0/0	0/0/1
Cecum [58]	1	68	0/1	1/0/0	1/0/0	0/0/1
Esophagogastric junction [59]	1	68	1/0	1/0/0	0/0/1	0/0/1

^a^The cases included in this table are those available in PubMed as of 5 August 2013. We grouped the cases that are expressed as mixed exocrine-endocrine carcinoma, mixed ductal-endocrine carcinoma and mixed acinar-endocrine carcinoma into a single mixed adenoneuroendocrine carcinoma (MANEC) category if they met the World Health Organization classification of endocrine tumors and the paper was published after 2010. We excluded the collision type of MANEC. *CGA* chromogranin A, *SYN* synaptophysin.

The clinical behavior of MANECs is still unclear due to the rarity of these tumors. In 2006, Volante *et al*. reported that the clinical behavior of MEECs follows that of most aggressive cell types [41]. In the present case, although the well-differentiated tubular adenocarcinoma was confined to the mucosa and submucosa, the NEC components had invaded the submucosa with vascular permeation. Therefore, we think that the pathological features of the NEC component will have a greater influence than those of the tubular adenocarcinoma on the prognosis of this patient.

The optimal treatment for esophageal MANEC has not yet been established. Basically, the standard treatment of patients with esophageal MANEC should be determined in accordance with the treatment recommended for esophageal squamous cell carcinoma. The resectability should be judged on the basis of the preoperative diagnosis, and the decision whether to provide adjuvant therapy should be made on the basis of the postoperative diagnosis. Preoperative chemotherapy is regarded as the standard treatment for patients with stage II/III esophageal squamous cell carcinoma in Japan [66]. In our patient, however, preoperative chemotherapy was not performed, because the preoperative diagnosis was stage IA and the histological diagnosis was not squamous cell carcinoma. Although surgery is the treatment of choice for limited disease of esophageal small cell carcinoma, defined as a tumor confined to a localized region, surgery alone has been found to lead to worse outcomes than adjuvant chemotherapy [67,68]. Investigators in several studies have reported that surgery could extend the survival time of patients with limited disease if it was performed as part of multimodal treatment [69,70]. Chemotherapy for esophageal NEC is usually administered according to the recommendations for chemotherapy for small cell lung cancer (SCLC) and usually consists of cisplatin and etoposide [68,69]. In our case, there was a choice regarding which adjuvant chemotherapy should be administered because of the pathological features of the NEC components representing vascular permeation. We did not administer adjuvant chemotherapy, however, because of the complicated postoperative course resulting from the bilateral recurrent laryngeal nerve injury, the fact that the stage of the disease was IA (pT1bN0M0) and the operation performed had sufficiently high curative potential. However, we think that close follow-up of the patient is mandatory. In addition, Noda *et al*. suggested the superiority of cisplatin and irinotecan over cisplatin and etoposide for metastatic SCLC [71], and cisplatin and irinotecan is another option for esophageal NEC in Japan.

## Conclusion

Our patient had a rare case of MANEC arising from EGM of the upper thoracic esophagus. To the best of our knowledge, this case report is the first of its kind published in the literature. Because the clinical behavior of esophageal MANEC is poorly understood, further accumulation of similar cases is necessary to clarify the optimal treatment for this disease.

## Consent

Written informed consent was obtained from the patient for publication of this case report and any accompanying images. A copy of the written consent is available for review by the Editor-in-Chief of this journal.

## Abbreviations

EGM: Ectopic gastric mucosa; MANEC: Mixed adenoendocrine carcinoma; NEC: Neuroendocrine carcinoma; SCLC: Small cell lung cancer; WHO: World Health Organization.

## Competing interests

The authors declare that they have no competing interests.

## Authors’ contributions

TK wrote the manuscript. SK, SL, SH, HS, MU and HU performed surgery. YO and TF carried out the pathological examination. KS, TF and HU were involved in the final editing. All authors read and approved the final manuscript.
